# A novel immunogenic mouse model of melanoma for the preclinical assessment of combination targeted and immune-based therapy

**DOI:** 10.1038/s41598-018-37883-y

**Published:** 2019-02-04

**Authors:** Emily J. Lelliott, Carleen Cullinane, Claire A. Martin, Rachael Walker, Kelly M. Ramsbottom, Fernando Souza-Fonseca-Guimaraes, Shatha Abuhammad, Jessica Michie, Laura Kirby, Richard J. Young, Alison Slater, Peter Lau, Katrina Meeth, Jane Oliaro, Nicole Haynes, Grant A. McArthur, Karen E. Sheppard

**Affiliations:** 1 Cancer Research Division, Peter MacCallum Cancer Centre, Melbourne, VIC Australia; 2 Sir Peter MacCallum Department of Oncology, University of Melbourne, Melbourne, VIC Australia; 3 Department of Medical Biology, University of Melbourne, Melbourne, VIC Australia; 4 Division of Molecular Immunology, The Walter Eliza Hall Institute of Medical Research, Parkville, VIC Australia; 5 Department of Pathology, Yale University School of Medicine, New Haven, CT USA; 6 Department of Pathology, University of Melbourne, Melbourne, VIC Australia; 7 Department of Medicine, St Vincent’s Hospital, University of Melbourne, Melbourne, VIC Australia; 8 Department of Biochemistry and Molecular Biology, University of Melbourne, Melbourne, VIC Australia

## Abstract

Both targeted therapy and immunotherapy have been used successfully to treat melanoma, but the development of resistance and poor response rates to the individual therapies has limited their success. Designing rational combinations of targeted therapy and immunotherapy may overcome these obstacles, but requires assessment in preclinical models with the capacity to respond to both therapeutic classes. Herein, we describe the development and characterization of a novel, immunogenic variant of the *Braf*^*V600E*^*Cdkn2a*^*−/−*^*Pten*^*−/−*^ YUMM1.1 tumor model that expresses the immunogen, ovalbumin (YOVAL1.1). We demonstrate that, unlike parental tumors, YOVAL1.1 tumors are immunogenic *in vivo* and can be controlled by immunotherapy. Importantly, YOVAL1.1 tumors are sensitive to targeted inhibitors of BRAF^V600E^ and MEK, responding in a manner consistent with human BRAF^V600E^ melanoma. The YOVAL1.1 melanoma model is transplantable, immunogenic and sensitive to clinical therapies, making it a valuable platform to guide strategic development of combined targeted therapy and immunotherapy approaches in BRAF^V600E^ melanoma.

## Introduction

The development of targeted therapies and immunotherapies in recent years has revolutionized the landscape of cancer treatment, particularly melanoma. The most notable clinical successes in melanoma include immune checkpoint inhibitors of PD-1 and CTLA-4^1–8^, and targeted inhibitors of the MAPK/ERK pathway; specifically dual inhibition of BRAF^V600E^ and MEK^9–15^. However, resistance to targeted therapies and low response rates to immunotherapies have prompted great interest in combining these therapeutic strategies. While combination therapies are now being evaluated in clinical trials, most are performed on the basis of observed clinical success of individual therapies, with limited understanding of how these therapeutic classes interact with one another. As such, little judgement can be made about optimal combinations and scheduling, or which patients to target with various combinations. Emerging evidence suggests that therapies targeting the MAPK/ERK pathway may also impact on anti-tumor immune responses^16–18^, and hence a thorough understanding of these interactions is paramount for the strategic design of efficacious targeted and immune therapy combinations.

The Yale University Mouse Melanoma (YUMM) series of cell lines can be efficiently grown and studied in immunocompetent C57BL/6 mice, and importantly, have been derived from genetically modified mice bearing mutations commonly found in human melanoma^19^. These models provide an immunocompetent and clinically relevant setting in which to study targeted and immune therapy combinations. However, as these lines were generated through the introduction of a small number of oncogenic driver mutations, they are poorly T cell immunogenic due to a low somatic mutational burden^20–22^; a major challenge for mouse models genetically engineered in this way^23,24^. Melanoma, in particular, is a highly mutated and immunogenic cancer^25^, expressing numerous neoantigens that have the capacity to stimulate strong immune responses^26–28^. The remarkable success of immunotherapies in the treatment of melanoma, in contrast to other solid cancers, is due in part to high inherent immunogenicity and acquired immunosuppressive mechanisms^29^. Hence, weakly immunogenic mouse models do not capture the full characteristics of human melanoma.

The YUMM1.1 line, derived from mice bearing a BRAF^V600E^ mutation and deficient for *Cdkn2a* and *Pten*, is poorly immunogenic due to low neoantigen expression, and resistant to immunotherapy due to low inflammatory and chemotaxis gene signatures^20–22^. In the present study we show that expression of ovalbumin (OVA) was sufficient to alter the susceptibility of YUMM1.1 tumors to host T cell mediated control. The adoptive transfer of OVA-specific CD8^+^ T cells (OT-I T cells), as well as immune checkpoint blockade therapy, further enhanced tumor control. Checkpoint inhibitors were ineffective against the parental YUMM1.1 model, indicating the expression of OVA, and enhanced T cell engagement, sensitizes this model to immunotherapy. Importantly, the response of this tumor line to standard-of-care BRAF and/or MEK inhibition was equivalent to that observed in human BRAF^V600E^ melanoma, consistent with the parental YUMM1.1 line^21^. Collectively, our data highlights the utility of YOVAL1.1 as a preclinical model for examining the complex interactions of targeted therapies and the immune system, providing a valuable platform to better guide clinical application of novel and existing therapy combinations in BRAF^V600E^ melanoma.

## Results

### Expression of the immunogen, ovalbumin, in YUMM1.1 tumor cells promotes T cell-mediated tumor control

**Figure 1:**
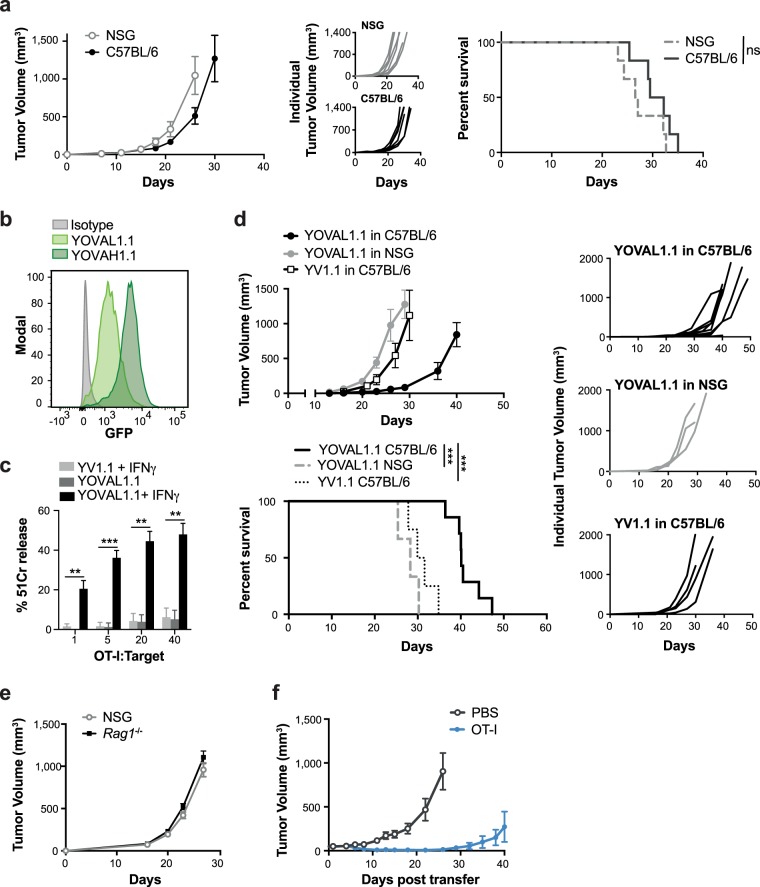
Expression of the immunogen, ovalbumin, in YUMM1.1 tumor cells promotes T cell-mediated tumor control. (**a**) Tumor growth and survival of 3 × 10^5^ YUMM1.1 cells in C57BL/6 mice or NSG mice, with survival measured as time for tumors to reach >1200 mm^3^. ns – not significant, log-rank (Mantel-Cox) test, n = 5–8. (**b**) YUMM1.1-OVA sorted by FACS into low and high GFP-expressing populations; YOVAL1.1 and YOVAH1.1, respectively. (**c**) Killing by OT-I T cells co-cultured for 4 hours at indicated ratios with 51Cr-labelled target cells pre-stimulated +/− IFNγ. One way ANOVA, Tukey’s multiple comparisons test, n = 3. (**d**) YOVAL1.1 tumor growth and survival in C57BL/6 mice or NSG mice with survival measured as time for tumors to reach >1200 mm^3^, log-rank (Mantel-Cox) test, n = 3–5. (**e**) Growth of YOVAL1.1 in NSG or *Rag1*^−/−^ mice, n = 3. (**f**) YOVAL1.1 tumor growth and survival following transfer of activated OT-I T cells or PBS. YV1.1 – YUMM1.1 transduced with empty vector. All error bars show ±SEM. **p < 0.01, ***p < 0.001.

The YUMM series of mouse melanoma cell lines are reported to be poorly T cell immunogenic *in vivo* due to low neoantigen expression^20–22^. Consistent with this, we found no significant difference in the growth kinetics or overall survival of YUMM1.1 tumors grown in immunocompetent C57BL/6 or immunodeficient NOD scid gamma (NSG) mice; which are T and B cell deficient and lack functional NK cells due to a null mutation in the IL-2 receptor common gamma chain (Fig. 1a). While these tumors induced the recruitment of IFNγ−producing NK cells (Supplementary Fig. 1a,b), this was not sufficient to control tumor growth. This was despite the fact that *in vitro*, NK cells could kill YUMM1.1 tumor cells and secrete IFNγ, which up-regulated MHC-I on the tumor cells (Supplementary Fig. 1c). Furthermore, while YUMM1.1 tumors express MHC I *in vivo* (Supplementary Fig. 1d) we speculate that, in the absence of sufficient neo-antigen expression on YUMM1.1 tumor cells, an anti-tumor T cell response was limited.

Human melanoma is inherently immunogenic due to a high neoantigen load^25^. To establish a model that would mimic the immunogenicity of melanoma, the YUMM1.1 line was retrovirally transduced to stably express OVA and GFP, and sorted for both low and high GFP expression (Fig. 1b). Both YUMM1.1 cells transduced with OVA, or an empty vector control (YV1.1), were resistant to OVA-specific OT-I T cell-mediated killing (Fig. 1c). However, pre-treatment of OVA-transduced YUMM1.1 tumor cells with IFNγ to induce H-2K^b^ expression and presentation of the OVA peptide SIINFEKL, sensitized them to OT-I T cell killing (Fig. 1c). OVA stimulates a strong CD8^+^ T cell response and when expressed at high levels on tumor cells, can prevent successful engraftment of tumors in C57BL/6 mice due to immune-mediated rejection^30^. Thus, we utilized the low OVA-expressing population for our *in vivo* studies, referred to here as YOVAL1.1 (**Y**UMM**1**.**1**-**OVA**-**L**ow).

We first examined the growth kinetics of the YOVAL1.1 tumor line in both C57BL/6 and NSG mice. Compared to that observed in NSG mice, growth of the YOVAL1.1 tumors was significantly slower in C57BL/6 mice, with a difference in median overall survival of 12 days (40 days versus 28 days; Fig. 1d). Notably, growth of the YV1.1 empty vector control tumors in C57BL/6 mice was comparable to YOVAL1.1 tumors grown in NSG mice (Fig. 1d). Furthermore, in *Rag1*^*−/−*^mice, which have a functional innate immune system but lack T and B cells, the YOVAL1.1 tumors grew out in a similar manner to that observed in NSG mice (Fig. 1e). YOVAL1.1 tumor growth in C57BL/6 mice was also significantly delayed following the adoptive transfer of OVA-specific OT-I T cells (Fig. 1f). Collectively these data support a role for T cells in mediating the control of YOVAL1.1 tumor growth *in vivo*.

### Expression of the immunogen, ovalbumin, in YUMM1.1 tumor cells alters the tumor immune microenvironment

**Figure 2:**
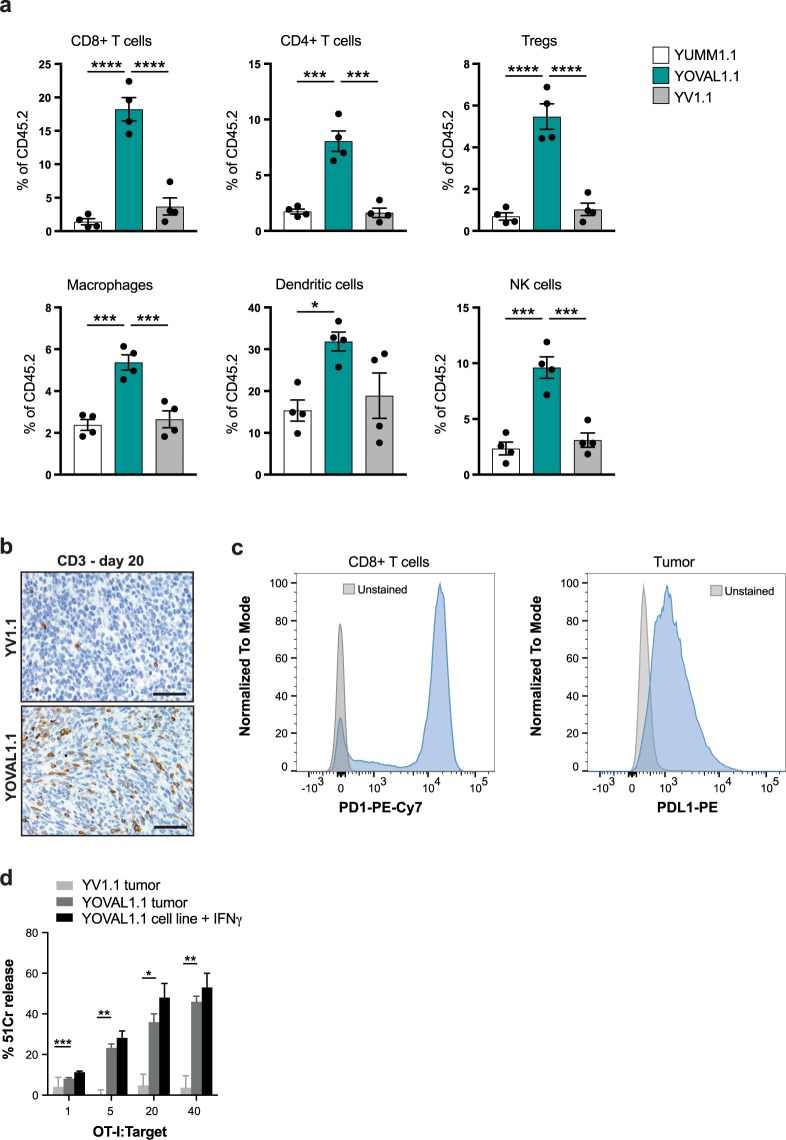
Expression of the immunogen, ovalbumin, in YUMM1.1 tumor cells alters the tumor immune microenvironment. Analysis of immune engagement following OVA introduction into YUMM1.1. (**a**) Flow cytometry analysis of immune infiltrate in YUMM1.1, YOVAL1.1 and YV1.1 tumors as a percentage of CD45.2^+^ cells, 4 weeks after implant. One way ANOVA, Tukey’s multiple comparisons test, n = 4. (**b**) Immunohistochemistry of CD3^+^ infiltrate in YV1.1 and YOVAL1.1 tumors, representative of n = 3. (**c**) PD-1 and PD-L1 expression on YOVAL1.1-infiltrating CD8+ T cells and YOVAL1.1 tumor cells, respectively, representative of n = 4. (**d**) Killing by OT-I T cells co-cultured for 4 hours at indicated ratios with 51Cr-labelled targets; YOVAL1.1 and YV1.1 *ex vivo* endpoint tumors (>1200 mm^3^) or YOVAL1.1 cells pre-stimulated with IFNγ. One way ANOVA, Tukey’s multiple comparisons test, n = 3. YV1.1 – YUMM1.1 transduced with empty vector. All scale bars show 50 μm. All error bars show ±SEM. *p < 0.05, **p < 0.01, ***p < 0.001, ****p < 0.0001.

To determine the impact of OVA expression on the tumor microenvironment, we compared the immune infiltrate in parental (YUMM1.1), empty vector control (YV1.1) and OVA-expressing (YOVAL1.1) tumors 4 weeks following implant. We observed a significant increase in the frequency of major immune subsets, including CD8^+^ T cells, CD4^+^ T cells, T regulatory cells, NK cells, dendritic cells and macrophages, within YOVAL1.1 tumors compared to control YUMM1.1 and YV1.1 tumors (Fig. 2a and Supplementary Figs 2–4). Indeed, immunohistochemical analysis of YOVAL1.1 tumors revealed markedly higher levels of infiltrating CD3^+^ T cells compared to YV1.1 tumors (Fig. 2b). Together these data indicate that YOVAL1.1 tumors can stimulate strong CD8^+^ T cell activity, which appears to contribute to immune-mediated tumor growth control in C57BL/6 mice. However, the induction of an anti-tumor T cell response was insufficient to cause complete tumor rejection, which may in part have been attributed to the observed increases in tumour associated T regulatory cell and/or macrophage frequency (Fig. 2a). Notably, expression of PD-1 and PD-L1 on the CD8^+^ TILs and YOVAL1.1 tumor cells, respectively, was also detected (Fig. 2c).

To confirm that YOVAL1.1 tumors did not escape immune control as a result of acquired resistance to T cell killing, we harvested tumors at endpoint (>1200 mm^3^) and found they were sensitive to killing by *in vitro* activated OT-I T cells (Fig. 2d). Collectively these observations suggest that therapies aimed at overcoming these immunosuppressive mechanisms, such as checkpoint blockade, may be effective in this model.

### YOVAL1.1 tumors are responsive to immune checkpoint blockade *in vivo*

**Figure 3:**
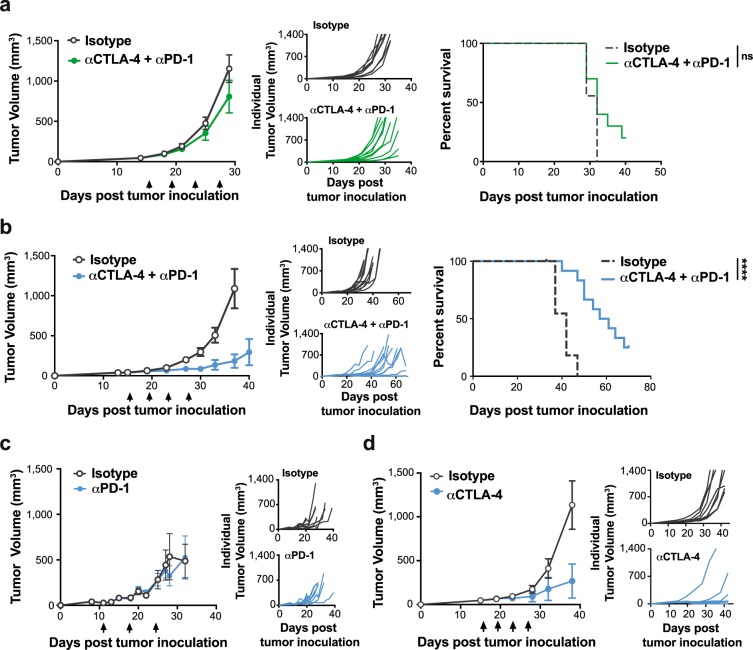
YOVAL1.1  tumors are responsive to immune checkpoint blockade *in vivo*. (**a**,**b**) Response of YUMM1.1 (**a**) and YOVAL1.1 (**b**) tumors to immunotherapy. Tumor growth and survival in response to combination anti-PD-1 and anti-CTLA-4 or isotype controls in C57BL/6 mice, n = 10–12. (**c**,**d**) YOVAL1.1 tumor growth in response to anti-PD-1 (**c**) or anti-CTLA-4 (**d**) or isotype controls. (**a**–**d**) Survival measured as time for tumors to reach >1200 mm^3^, arrows indicate treatment days. ns – not significant, Log-rank (Mantel-Cox) test, error bars show ±SEM. ****p < 0.0001.

To determine if potential immunosuppressive mechanisms could be overcome in this model, YOVAL1.1 tumor-bearing C57BL/6 mice were treated with antibodies to the checkpoint blockade receptors PD-1 and CTLA-4. The combination of anti-PD-1 and anti-CTLA-4 checkpoint blockade is a standard-of-care therapy approach in melanoma^4–6^. Analysis of this combination strategy in C57BL/6 mice bearing parental YUMM1.1 tumors revealed that this model is poorly responsive, with no significant improvement in median survival relative to isotype control treated mice (Fig. 3a). However, we hypothesized that the enhanced capacity of YOVAL1.1 tumors to trigger an endogenous T cell response would correlate with improved response to immunotherapy.

To directly compare the two models, which grow with different kinetics *in vivo*, we inoculated mice with 2 × 10^6^ YOVAL1.1 cells or 1.5 × 10^6^ YUMM1.1 cells to establish tumors of the same size at day 15 post inoculation; the time at which treatment was started (Supplementary Fig. 5). In contrast to YUMM1.1 tumors, there was a significant extension of survival of YOVAL1.1-bearing C57BL/6 mice co-treated with anti-PD-1 and anti-CTLA-4 therapy compared to isotype controls, with median survival of 57 days versus 40 days post tumor inoculation, respectively (Fig. 3b). Significantly, 10/12 mice treated with the combination therapy were still alive 50 days post tumor injection, compared to 0/12 mice in the control group. Notably, the YOVAL1.1 tumors were resistant to anti-PD-1 monotherapy (Fig. 3c), but responsive to anti-CTLA-4 monotherapy (Fig. 3d), indicating that the response to the combination therapy was predominately anti-CTLA-4 driven. The immunogenicity of YOVAL1.1 tumors, and the capacity of these tumors to respond to immunotherapy *in vivo*, makes this a valuable model to dissect how these immunotherapy approaches may be enhanced by target therapies.

### The response of YOVAL1.1 tumors to MAPK/ERK pathway-targeted therapy recapitulates that of human models

**Figure 4:**
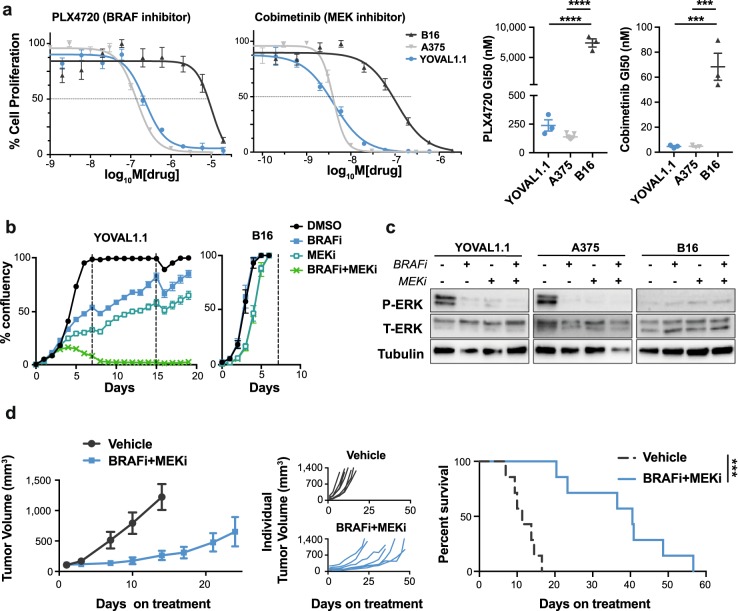
YOVAL1.1 is responsive to MAPK/ERK pathway-targeted therapy. Response of YOVAL1.1 to BRAF and MEK inhibition (BRAFi and MEKi). (**a**) Cell proliferation of YOVAL1.1, B16 and A375 cultured in increasing concentrations of PLX4720 (BRAFi) or cobimetinib (MEKi) for 5–6 days. Representative dose response curve shown. GI50s (right panels) were calculated as the concentration of drug leading to 50% growth inhibition. One-way ANOVA, Tukey’s multiple comparisons test, n = 3. (**b**) YOVAL1.1 or B16 cells cultured with DMSO, 1 μM PLX4720 (BRAFi), 10 nM cobimetinib (MEKi) or BRAFi + MEKi and cell confluency measured every 12–24 hours using IncuCyte®ZOOM. Representative graph of n = 3. (**c**) Western blot of phospho- and total-ERK (P-ERK and T-ERK) expression in YOVAL1.1, A375 and B16 treated with DMSO, 1 μM PLX4720 (BRAFi), 10 nM cobimetinib (MEKi) or BRAFi + MEKi for 48 hours, representative blot of n = 3. All images were cropped from different parts of the same blot, with exposure times differing according to antibody and cell line (see Supplementary Fig. 7). (**d**) Tumor growth and survival in YOVAL1.1-bearing C57BL/6 mice treated daily, 6 days/week, with dabrafenib (BRAFi) plus trametinib (MEKi). Survival is measured as time for tumors to reach >1200 mm^3^, log-rank (Mantel-Cox) test, n = 7. All error bars show ±SEM. ***p < 0.001, ****p < 0.0001.

The YUMM series of cell lines were developed through the introduction of common mutations observed in human melanoma^21^. Specifically, YUMM1.1 has a BRAF^V600E^ mutation and is sensitive to treatment with BRAF inhibitors (BRAFi) *in vitro*^21^. However, the response of YUMM1.1 to BRAFi, and standard-of-care therapy BRAFi plus MEKi, has not been reported *in vivo*, nor has the sensitivity to these inhibitors been compared to other common melanoma preclinical models. Hence, to determine the suitability of YOVAL1.1 as a model for studying targeted therapy, we examined its response to inhibitors of BRAF^V600E^ and MEK. As a measure of sensitivity, we determined drug doses of PLX4720 (BRAF^V600E^ inhibitor) and cobimetinib (MEK inhibitor) required for 50% growth inhibition (GI50). The PLX4720 and cobimetinib GI50s of YOVAL1.1 were not significantly different to those of the human BRAF^V600E^ melanoma line, A375 (239 ± 50 nM and 4.6 ± 0.7 nM vs. 139 ± 9 nM and 4.7 ± 0.5 nM, respectively) (Fig. 4a). In contrast, B16 cells, which lack a clinically relevant genetic background, were not sensitive to these inhibitors (PLX4720 and cobimetinib GI50s 7,412 ± 675 nM and 68.3 ± 10.8 nM, respectively) (Fig. 4a).

In melanoma patients and in preclinical models using human xenografts, combined inhibition of BRAF and MEK achieves synergistic anti-cancer responses^9,12–14^. Importantly, synergy between PLX4720 and cobimetinib was also observed in the YOVAL1.1 model. *In vitro* the combination synergistically halted the proliferative activity of the YOVAL1.1 line, whereas no such synergy was observed in the B16 line (Fig. 4b). PLX4720, cobimetinib and their combination decreased P-ERK levels in YOVAL1.1 and A375 cells, but not in the non-sensitive B16 line (Fig. 4c). *In vivo*, the combination of BRAF and MEK inhibition significantly improved survival of YOVAL1.1-bearing C57BL/6 mice, with a median overall survival of 40 days on treatment, compared to 12 days for vehicle-treated mice (Fig. 4d), which was similar to the response of the parental YUMM1.1 line (Supplementary Fig. 6). Taken together, these data demonstrate that YOVAL1.1 tumors respond to MAPK/ERK pathway inhibition with similar sensitivity to that of human preclinical models. This highlights the utility of YOVAL1.1 tumors as a clinically relevant *in vivo* model for studying responses to these targeted therapies in combination with immune-based approaches.

## Discussion

Immunotherapy and targeted therapy have both been immensely successful in extending the life of melanoma patients. However, the majority of patients treated with targeted therapy eventually relapse and approximately 50–70% of patients treated with immune checkpoint therapy do not respond^6,10–13,15^. In addition to inhibiting tumor intrinsic growth pathways, it is now well known that targeted therapies also impact anti-cancer immune responses^16–18^. Understanding these interactions is paramount in the strategic design of immune and targeted therapy combinations and this requires physiologically relevant, preclinical models that are both immunogenic, and responsive to standard-of-care therapies. Here, we describe YOVAL1.1 as a novel mouse model of melanoma that is suitable for evaluating *in vivo* immune interactions in response to targeted therapy.

Chicken ovalbumin (OVA) is widely used as a model antigen in T cell biology. Numerous mouse cancer models, including the commonly used B16 melanoma cell line, have been modified to express OVA to aid in enhancing and tracking tumor-specific T cell responses^31,32^; however, these models lack the genetic background commonly found in human melanoma. In contrast, the recently developed, YUMM series of cell lines carry the relevant genetic background and are fast becoming the preferred syngeneic model of melanoma^20–22,33^. In this study, we have introduced OVA into YUMM1.1 cells, to enhance *in vivo* immune interactions and thus have created a melanoma OVA model antigen system that is more clinically applicable.

Low immunogenicity is a known major challenge for genetically engineered mouse models^23,24^. Indeed, we found YUMM1.1 to be poorly immunogenic *in vivo*. The introduction of OVA to generate the YOVAL1.1 cell line was sufficient to sensitize the line to endogenous T cell control, but did not cause complete tumor rejection, supporting the presence of immunosuppressive mechanisms in this model. Consistent with this, we found an abundance of regulatory T cells within these tumors, in addition to expression of the immunosuppressive checkpoint molecules, PD-1 and PD-L1, on the T cells and tumor cells respectively. The loss of PTEN in this model is also a likely contributor to such strong immunosuppression, as PTEN loss in melanoma is associated with increased production of immunosuppressive cytokines and resistance to T cell-mediated immunotherapies^34–36^. Interestingly, the enhanced immunogenicity of YOVAL1.1 rendered the model amenable to checkpoint blockade with anti-CTLA-4, but not anti-PD-1, despite PD-1 and PD-L1 being expressed in the microenvironment, and anti-PD-1 demonstrating superior results to anti-CTLA-4 in the clinic^37^. This observed resistance to PD-1 blockade therapy is comparable to observations reported previously in a YUMM model with the same genetic background^22^. Recently, the combination of anti-CTLA-4 and anti-PD-1 was shown to be superior to anti-PD-1 monotherapy^6^, and it is currently unclear whether this added benefit is due to complementary actions of the inhibitors, or a subset of anti-PD-1-resistant patients who are responsive to anti-CTLA-4. Our data suggests the latter is possible, given that this model responds equally well to anti-CTLA-4 with, and without, the addition of anti-PD-1. The primary anti-tumor mechanism of CTLA-4 checkpoint blockade remains controversial. In addition to enhancing T cell priming through blockade of inhibitory interactions between antigen presenting cells and T cells^38^, anti-CLTA-4 therapy may also deplete T regulatory FOXP3^+^ cells in the tumor microenvironment^39–41^. Importantly, this model provides a unique platform to dissect these mechanisms, which may provide insight into which patients are most likely to respond to anti-CTLA-4 therapy alone. Conversely, the innate resistance of the model to anti-PD-1 therapy may offer insight into mechanisms contributing to such resistance. Given the high toxicity^42^ and significant monetary costs^43^ of combined immune checkpoint therapies, there is great value in stratifying patients who will receive benefit from single agents or novel combination approaches.

Inhibition of BRAF and MEK is standard-of-care targeted therapy for BRAF^V600E^ melanoma, and the clinical response rate of BRAF^V600E^ melanoma to combined BRAF/MEK inhibition is around 70%^44^. However, there is now substantial evidence that these inhibitors alter anti-tumor immune responses^16–18^, potentially impacting on the efficacy of immunotherapy. To comprehensively evaluate the effects of these inhibitors on the immune system using preclinical models, it is essential that the model system is sensitive to these therapies. The response of YOVAL1.1 to BRAF and MEK inhibition recapitulates that of human BRAF^V600E^ A375 melanoma, demonstrating its suitability over the commonly used B16 syngeneic melanoma model. Importantly, these drugs demonstrated target specificity in YOVAL1.1, as evidenced by a reduction in P-ERK, and markedly decreased tumor progression *in vivo*. The expression of OVA in this model make it an ideal platform to evaluate the effects of these inhibitors on anti-tumor endogenous T cell responses, as well as adoptively transferred OVA-specific T cells.

In addition to being immunogenic and sensitive to clinical therapies, YOVAL1.1 tumors are transplantable, making the model a simple and effective tool to trial a range of novel and existing therapy combinations and scheduling. We therefore propose that the YOVAL1.1 melanoma model will provide a valuable platform to guide strategic development of combined targeted therapy and immunotherapy approaches in BRAF^V600E^ melanoma.

## Methods

### Cell culture and IFNγ stimulation

YUMM1.1, YOVAL1.1 and YV1.1 were cultured in RPMI 1640 plus 20 mM HEPES containing 10% FBS, 1% GlutaMAX, 1 mM Sodium Pyruvate, 1 mM MEM Non-Essential Amino Acids and 0.1% 2-mercaptoethanol. B16 and PhoenixE cells were cultured in DMEM containing 10% FBS and 1% GlutaMAX. A375 were cultured in RPMI 1640 plus 20 mM HEPES containing 10% FBS and 1% GlutaMAX. For IFNγ stimulation, cells were cultured in 2 ng/mL recombinant mouse IFNγ for 16 hours and washed twice with PBS prior to use in assays. All cell lines were harvested using Trypsin-EDTA (0.25%). Splenocytes were cultured in RPMI 1640 plus 20 mM HEPES containing 10% FBS, 1% GlutaMAX, 1 mM Sodium Pyruvate, 1 mM MEM Non-Essential Amino Acids, 0.1% 2-mercaptoethanol and Antibiotic-Antimitotic. All cells were cultured at 37 °C in 5% CO_2_.

### Ovalbumin transduction

For virus production, PhoenixE cells were transfected with MSCV-IRES-GFP-OVA or MSCV-IRES-GFP in polyethylenimine (PEI), using 1 μg DNA/4.5uL PEI/mL PhoenixE media. After 16 hours, viral media was collected and combined 1:1 with YUMM1.1 media. Media was supplemented with 10 ng/mL protamine sulphate, and an additional 5% FBS before being added to YUMM1.1 cells. This was repeated three times over 20 hours. Transduced YUMM1.1 cells were sorted for GFP expression using BD FACS Aria II (BD Biosciences, North Ryde, New South Wales, Australia).

### Primary T and NK cell isolation and activation

Spleens from C57BL/6.OT-I transgenic mice were filtered through a 70 μM filter and red blood cells were lysed with red cell lysis buffer (150 mM NH_4_Cl, 10 mM KHCO_3_, 0.1 mM Na_2_EDTA). OT-I T cells were activated by culturing with 10 nM SIINFEKL plus 100 IU/mL IL-2 for 72 hrs, and then washed and cultured with 75 IU/mL IL-2 for a further 3–6 days prior to use in assays or for adoptive transfer studies. NK cells were isolated from spleens of C57BL/6 mice using a mouse NK cell enrichment kit (EasySep^TM^ #19755) and cultured in 250 IU/mL IL-2 for 5–7 days prior to use in assays.

### Tumor cell preparation

Tumors were digested with Collagenase IV (1.6 mg/mL) and DNase (2 U/mL) in DMEM for 45 minutes at 37 °C with agitation and filtered through a 70 μM filter to make a single cell suspension. Tumor preparations were sorted for GFP^+^ cells using BD FACS Aria II.

### Cytotoxic assays

Target cells were labelled with 250 μ*Ci*/mL Chromium-51 (51Cr; Perkin Elmer, VIC, Australia) for 45 minutes prior to culturing with effector cells at 37 °C. Supernatant was collected and 51Cr was measured by a gamma counter (Wallac Wizard). 51Cr release due to effector-mediated killing was calculated as *%Release* = *[(51Cr*_*SAMPLE*_ − *51Cr*_*SPONT*_*)/(51Cr*_*TOTAL*_ − *51Cr*_*SPONT*_*)]* × 100; where 51Cr_SPONT_ is spontaneous 51Cr release from target cells cultured without effector cells, and 51Cr_TOTAL_ is total chromium release from cells lysed with 10% Triton X-100.

### Flow cytometry

Samples were blocked with 2% normal mouse serum or mouse Fc block (2.4G2, BD Biosciences). Fixable yellow (Invitrogen, L34959) was used to stain live/dead cells. Anti-mouse antibodies used were CD45.2 (104, Tonbo Biosciences), CD3 (500A2, BD Bioscience), TCRβ (H57–597, eBioscience), CD8 (53-6.7, BioLegend), CD4 (GK1.5, BioLegend), FoxP3 (FJK-16S, eBioscience), Ly6C (HK1–4, BioLegend), CD11b (M1/70, BioLegend), F4/80 (BM8, eBioscience), MHC II (M5/114.15.2, eBioscience), CD11c (N418, BioLegend), NK1.1 (PK136, BD Biosciences), IFNγ (XMG1.2, Tonbo Biosciences), H-2Db (28-14-8, eBioscience), H-2Kb (AF6–88.5.5.3, eBioscience), PD-1 (29 F.1A12, BioLegend), and PD-L1 (MIH5, eBioscience). Fluorescence was measured on BD LSR Fortessa^TM^ X-20 or BD FACSymphony^TM^ flow cytometer (BD Biosciences, North Ryde, New South Wales, Australia) and data analysed using FlowJo, LLC software.

### Cytokine bead array

IFNγ was measured with a mouse CBA inflammation kit (BD Biosciences, 552364) according to manufacturer’s instructions. Samples were analyzed using a FACS Verse (BD Biosciences, North Ryde, New South Wales, Australia).

### Immunohistochemistry

Tumors were fixed with 10% neutral buffered formalin and paraffin embedded. 4 μm tumor slices were immunostained with anti-CD3 (SP7, Abcam, used at 1:600) following a standard IHC protocol and utilising a DAKO Autostainer (Agilent Technologies). Slides were imaged on an Olympus BX51 microscope.

### *In vivo* mouse experiments

All animal studies were performed in accordance with the NHMRC Australian code for the care and use of animals for scientific purposes 8^th^ edition (2013) and with approval from the Peter MacCallum Animal Experimentation Ethics Committee or the Walter and Eliza Hall and Harry Perkins Institute’s Animal Ethics Committees. Male C57BL/6 and *Rag1*^*−/−*^ mice were purchased from Walter Eliza Hall Institute and NOD scid gamma (NSG) mice were bred in-house. 6–10 week old mice were shaved 1–2 days prior to tumor implants and anaesthetised for injections. Mice were injected subcutaneous on the right flank with 2 × 10^6^ cells in 100 μL PBS using a 27 G needle. Tumors were measured 2–3 times/week and mice were euthanized when tumor volume reached >1200 mm^3^. For all therapy studies, mice were randomised according to tumor size on the day that therapy commenced. For targeted therapies, dabrafenib (30 mg/kg; Selleckchem, Houston, TX) plus trametinib (0.3 mg/kg; HY-10999 Focus Bioscience) were co-administered by a single daily oral gavage (vehicle 0.5% hydroxypropylmethyl cellulose, 0.2% Tween 80 in H_2_O), 6 days/week, starting when tumors reached 50–200 mm^3^. For immunotherapies, anti-PD-1 (RMP1–14) and anti-CTLA-4 (9H10), or corresponding isotypes Rat IgG2a and Syrian Hamster, respectively, were purchased from Bio X Cell (West Lebanon, NH) and administered by intraperitoneal injection. For anti-CTLA4 monotherapy and combination anti-PD-1 plus anti-CTLA-4, 200 μg/150 μg of anti PD-1/CTLA-4 or corresponding isotypes was administered to each mouse on day 15 post tumor inoculation, followed by 3 further doses of 150 μg/100 μg, 4 days apart. For anti-PD-1 monotherapy, 200 μg of anti-PD-1 or isotype was administered once per week for 3 weeks starting 11 days post tumor inoculation. For OT-I T cell transfer, mice were given 4 Gy total body irradiation (using X-RAD iR-160; Precision X-Ray, North Branford, CT) on day 16 post tumor inoculation, followed by intravenous administration of 1 × 10^7^ primary OT-I T cells. Mice were given 50,000 IU IL-2 by intraperitoneal injection daily for 5 days post OT-I T cell transfer.

### *In vitro* drug response

Cells were cultured in PLX4720 (Euroasian Chemicals, Mumbai, India) or cobimetinib (Selleckchem, Houston, TX, USA). For dose response curves, cells were cultured in drug for 5–6 days, then fixed with 100% methanol followed by 20 minutes in 2 N HCl + 0.5% Triton X-100, 10 minutes in 0.1 M Na_2_B_4_0_7_.H_2_0 (pH 8.5) and nuclear stained with 1 μg/mL propidium iodide. Cells were counted by Red Object Count using the Incucyte Zoom® (Essen BioScience). For proliferation assays, confluency was measured every 12–24 hours using the Incucyte Zoom®.

### Western blot

Cells were lysed in buffer containing 0.5 mM EDTA, 20 mM HEPES and 2% SDS (pH 7.9), incubated at 95 °C for 10 minutes and passed through a 25 G syringe. Protein was quantified with DT^TM^ protein kit (Bio-Rad #5000111), run on 4–20% SDS-PAGE gel and transferred by BioRad semi-dry transfer system. Immobulin-P polyvinylidene fluoride (PVDF) membrane (Millipore) was blocked with 5% skim milk in Tris-buffered saline plus 0.1% Tween-20 prior to probing with primary antibodies and HRP-conjugated secondary antibodies. ECL western blotting detection kit (GE Healthcare #45000878) was used for detection. Primary antibodies used were p44/42 (Erk1/2) Rabbit pAb (#9102 S) and phospho-p44/42 MAPK (Erk1/2) (Thr202/Tyr204) (D13.14.4E) XP® Rabbit mAb (#4370), both used at 1:1000.

### Statistical analysis

One-way analysis of variance (ANOVA) with Tukey’s multiple comparisons tests and unpaired *t-*tests were performed using GraphPad PRISM. Kaplan-Meier survival was compared using a log-rank (Mantel-Cox) test. All experiments were performed in at least three biological replicates. Error bars show ±SEM. Significance was determined as *p < 0.05, **p < 0.01, ***p < 0.001, ****p < 0.0001.

## Supplementary information

Supplementary Figures

## Data Availability

All data generated or analysed during this study are included in this published article (and its Supplementary Information files).
