# Supplementary material for: A novel immunogenic mouse model of melanoma for the preclinical assessment of combination targeted and immune-based therapy
Source: Sci Rep. 2019 Feb 4;9:1225. doi: 10.1038/s41598-018-37883-y (PMC6361951; doi:10.1038/s41598-018-37883-y)
Supplement: Supplementary file 1 — Supplementary Figures [file 41598_2018_37883_MOESM1_ESM.pdf]

Supplementary Information

A novel immunogenic mouse model of melanoma for the preclinical assessment of combination targeted and immune-based therapy

Emily J. Lelliott[1,2], Carleen Cullinane[1,2], Claire A. Martin[1], Rachael Walker[1], Kelly M. Ramsbottom[1], Fernando Souza-Fonseca-Guimaraes[3,4], Shatha Abuhammad[1], Jessica Michie[1,2], Laura Kirby[1], Richard J. Young[1], Alison Slater[1], Peter Lau[1,2], Katrina Meeth[5], Jane Oliaro[1,2], Nicole Haynes[1,2,6], Grant A. McArthur[1,2,7], Karen E. Sheppard*[1,2,8]

[1]Cancer Research Division, Peter MacCallum Cancer Centre, Melbourne, VIC, Australia

[2]Sir Peter MacCallum Department of Oncology, University of Melbourne, Melbourne, VIC, Australia

[3]Department of Medical Biology, University of Melbourne, Melbourne, VIC, Australia

[4]Division of Molecular Immunology, The Walter Eliza Hall Institute of Medical Research, Parkville, VIC, Australia

[5]Department of Pathology, Yale University School of Medicine, New Haven, CT, USA

[6]Department of Pathology, University of Melbourne, Melbourne, VIC, Australia

[7]Department of Medicine, St Vincent's Hospital, University of Melbourne, Melbourne, VIC, Australia

[8]Department of Biochemistry and Molecular Biology, University of Melbourne, Melbourne, VIC, Australia

*Corresponding Author: Karen E. Sheppard

Email: karen.sheppard@petermac.org; Phone: +61385596681

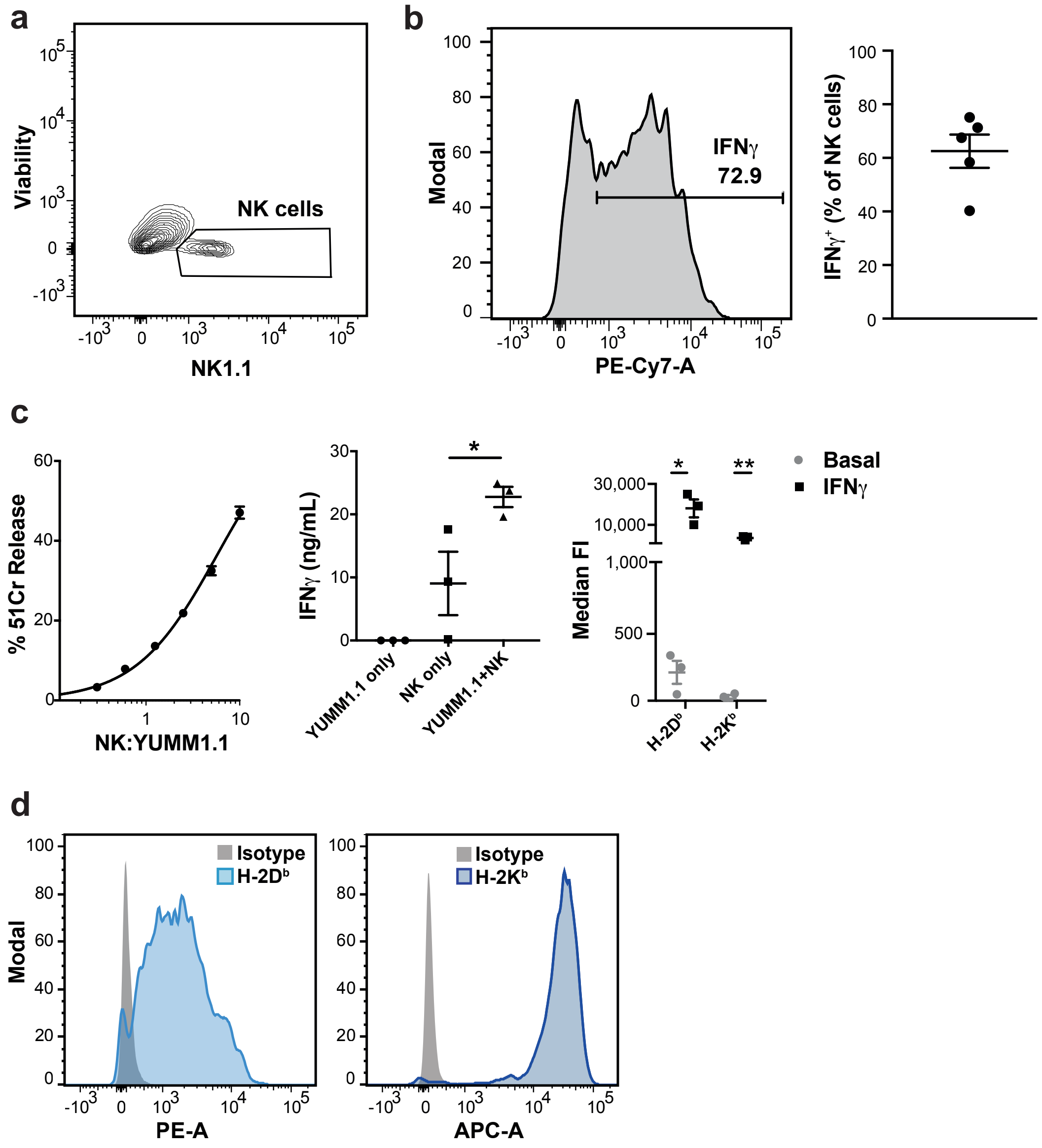

Supplementary Figure 1. a-b, NK cells **(a)** and IFNγ-expressing NK cells **(b)** in YUMM1.1 tumors harvested from C57BL/6 mice, plots representative of n=5. **c,** Left panel - Killing by splenic-derived NK cells co-cultured for 4 hours with 51Cr-labelled YUMM1.1 cells at indicated ratios, representative of n=3. Middle panel - IFNγ secretion by splenic-derived NK cells co-cultured for 4 hours with YUMM1.1 cells at a ratio of 2:1, IFNγ secretion was measured by cytokine bead array, One way ANOVA, Tukey's multiple comparisons test, n=3. Right panel - MHC I expression on unstimulated (basal) and IFNγ-stimulated YUMM1.1 cells in vitro, unpaired t test, n=3. **d,** MHC I expression on YUMM1.1 tumors harvested from C57BL/6 mice, representative of n=3. All error bars show ±SEM. *p<0.05, **p<0.01.

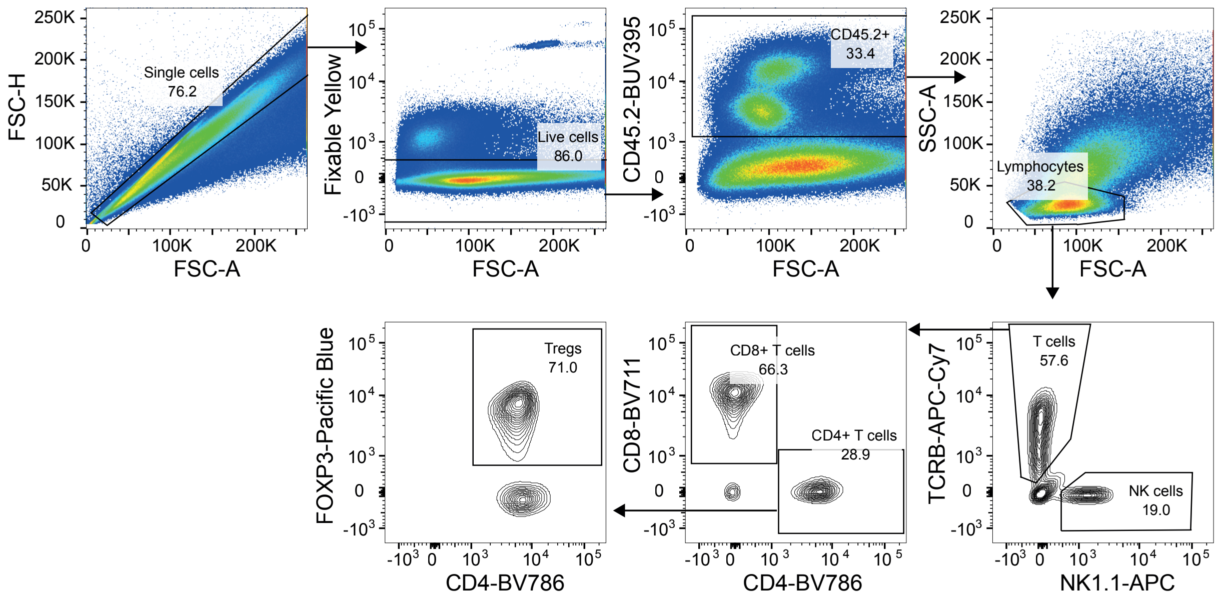

Supplementary Figure 2. Gating strategy on tumors for defining infiltrating CD8+ T cells, CD4+ T cells, regulatory T cells (Tregs) and NK cells.

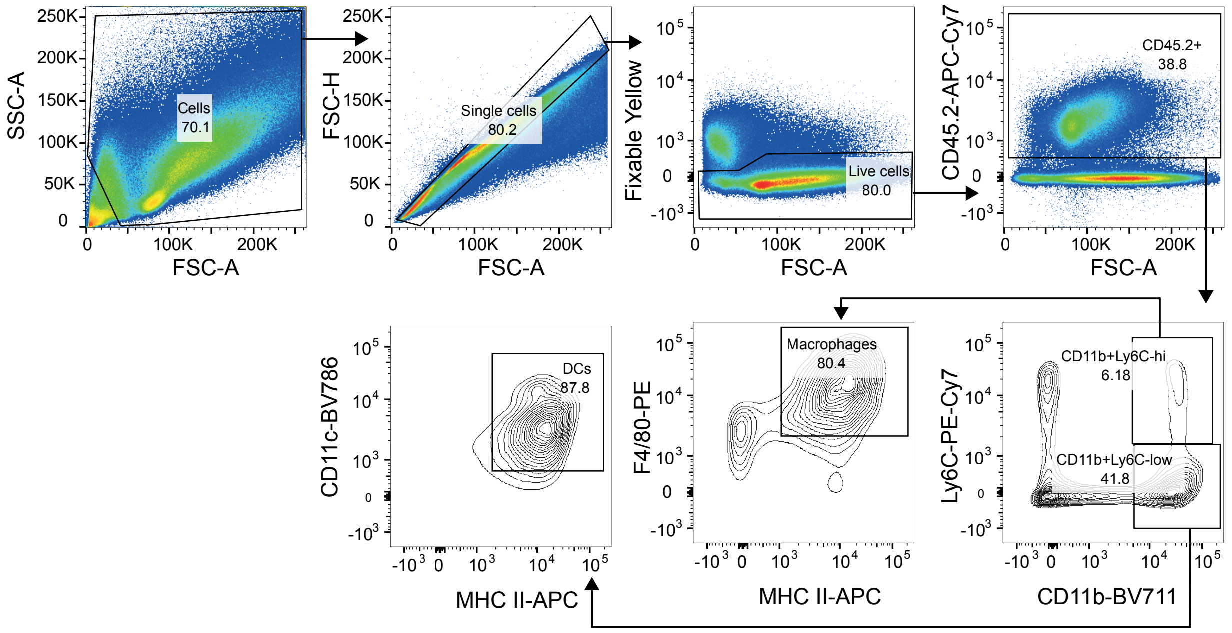

Supplementary Figure 3. Gating strategy on tumors for defining infiltrating macrophages and dendritic cells (DCs).

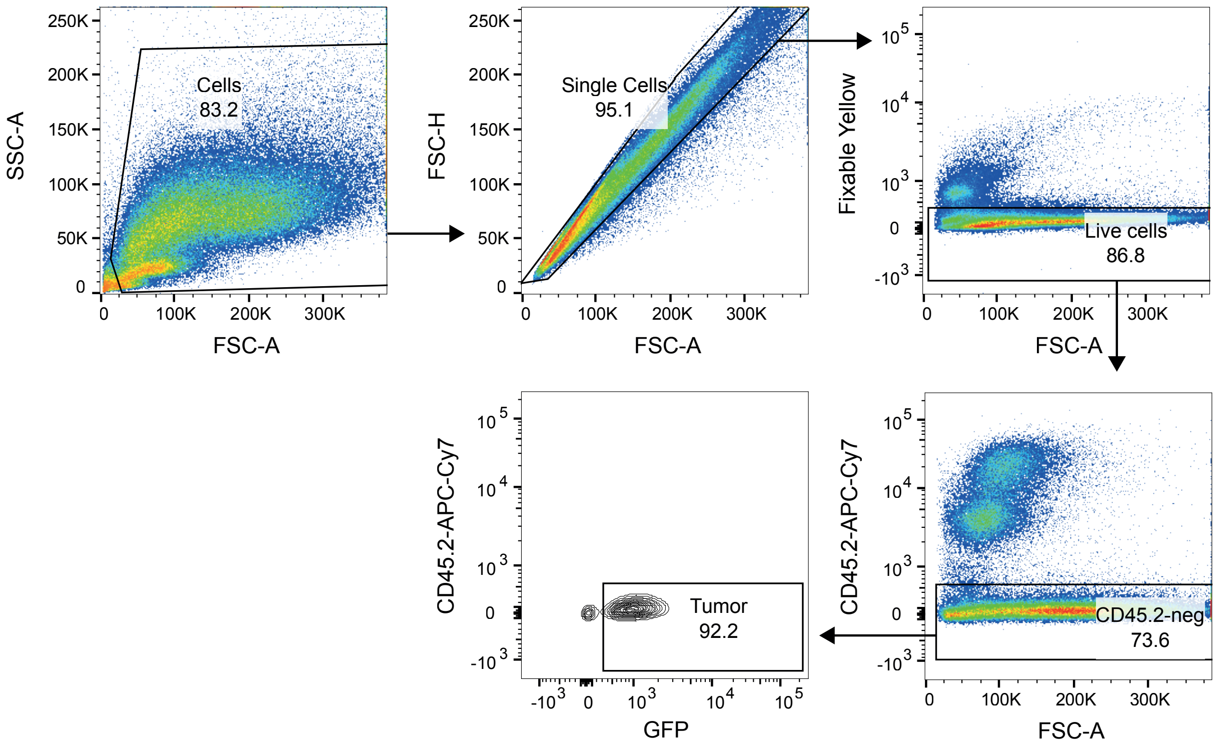

Supplementary Figure 4. Gating strategy for YOVAL1.1 tumor cells.

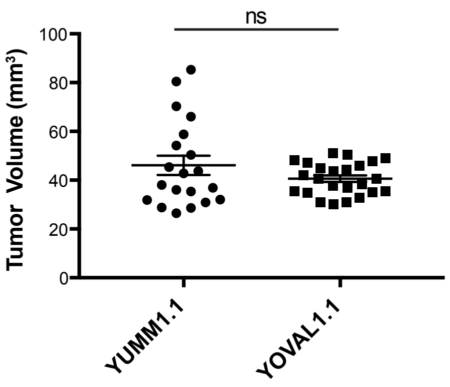

Supplementary Figure 5. Tumor volume at day 15 when immunotherapies were commenced. ns - not significant, unpaired t test, n=20-24. Error bars show ±SEM.

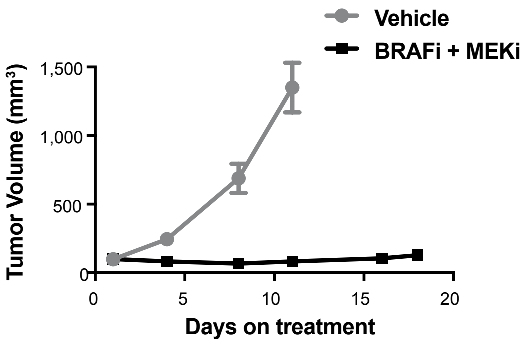

Supplementary Figure 6. YUMM1.1 tumor growth in C57BL/6 mice treated daily, 6 days/week, with dabrafenib (BRAFi) plus trametinib (MEKi). Error bars show ±SEM.

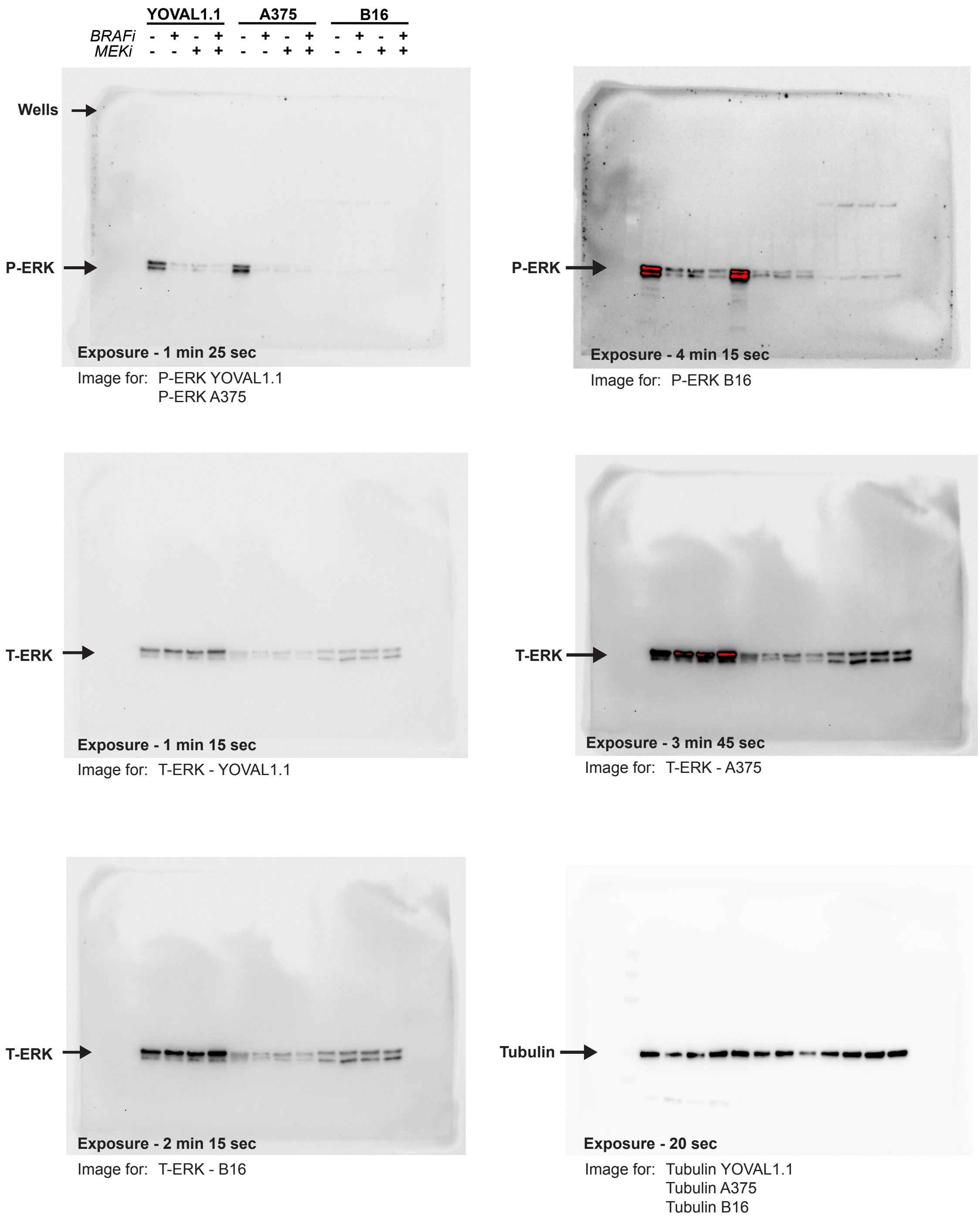

Supplementary Figure 7. Full-length blots used to compile Figure 4c. All images were cropped from different parts of the same blot at indicated exposure times. P-ERK - phosp-ERK; T-ERK - Total-ERK.
